# Retraction: ErbB4 preserves blood-brain barrier integrity via the YAP/PIK3CB pathway after subarachnoid haemorrhage in rats

**DOI:** 10.3389/fnins.2025.1642419

**Published:** 2025-06-13

**Authors:** Frontiers Editorial Office

The publisher retracts the article cited above.

Following publication, concerns were raised regarding the integrity of the images in the published figures. The authors failed to provide a satisfactory explanation during the investigation, which was conducted in accordance with Frontiers' policies.

This retraction was approved by the Chief Executive Editor of Frontiers. The authors received communication regarding the retraction and had a chance to respond. This communication has been recorded by the publisher.

Frontiers would like to thank Rene Aquarius for contacting the journal regarding the published article.

